# Clinical characteristics and prognostic analysis of SMARCA4‐deficient non‐small cell lung cancer

**DOI:** 10.1002/cam4.6083

**Published:** 2023-05-15

**Authors:** Xiyue Liang, Xianzheng Gao, Feng Wang, Shenglei Li, Yashu Zhou, Peng Guo, Yuanyuan Meng, Taiying Lu

**Affiliations:** ^1^ Department of Oncology The First Affiliated Hospital of Zhengzhou University Zhengzhou China; ^2^ Department of Pathology The First Affiliated Hospital of Zhengzhou University Zhengzhou China

**Keywords:** BRG1, *EGFR* mutation, lung cancer, prognosis, SMARCA4

## Abstract

**Purpose:**

To improve the understanding of special types of tumors, we summarized and analyzed the clinicopathological features and prognostic factors of SMARCA4‐deficient non‐small cell lung cancer (SMARCA4‐dNSCLC).

**Methods:**

We selected 105 patients with SMARCA4‐dNSCLC and 221 patients with SMARCA4‐intact non‐small cell lung cancer (SMARCA4‐iNSCLC) by performing immunohistochemical analysis of 1520 NSCLC samples, and we assessed the patients' clinicopathological features and survival state.

**Results:**

(1) SMARCA4‐dNSCLC was significantly associated with older age, male sex, smoking history, larger invasive tumor size, higher tumor proliferation index (Ki‐67), more adrenal metastases, more lymph node metastases, and few *EGFR* mutations (*p* < 0.05). The tumors were mostly negative for thyroid transcription factor‐1 (TTF‐1), CD34, and p40 and positive for cytokeratin 7 (CK7) in immunohistochemistry (IHC). Nineteen SMARCA4‐dNSCLC patients mostly had *TP53*, *SMARCA4*, and *LRP1B* mutations, and 48% of them had *SMARCA4* frameshift mutations. SMARCA4‐dNSCLC patients have a worse prognosis than SMARCA4‐iNSCLC patients (HR: 0.27; 95% CI: 0.17–0.45). The overall survival (OS) of patients with stage III SMARCA4‐dNSCLC was worse than that of patients with SMARCA4‐iNSCLC, and the OS of stage IV SMARCA4‐dNSCLC patients was also worse than that of SMARCA4‐iNSCLC patients (*p* < 0.01). (2) Multivariate regression analysis showed that sex (HR: 4.12; 95% CI: 1.03–16.39) and smoking history (HR: 2.29; 95% CI: 1.04–5.02) had significant effects on the survival time of SMARCA4‐dNSCLC patients. In SMARCA4‐dNSCLC patients without distant metastases (stage I–III), patients with stage N2 or N3 lymph node metastases (HR: 6.35; 95% CI: 1.07–37.47) had a poor prognosis. Among patients with SMARCA4‐dNSCLC who were treated and had distant metastases (stage IV), male patients and patients treated with immunotherapy combined with chemotherapy showed a longer median overall survival (mOS).

**Conclusion:**

SMARCA4‐dNSCLC has unique clinicopathological features and a shorter survival prognosis than SMARCA4‐iNSCLC. The efficacy of immunotherapy combined with chemotherapy needs to be observed for longer periods.

## 1 INTRODUCTION

Since the World Health Organization (WHO) released its thoracic tumor classification system in 2021, many molecules affecting tumor progression have been identified as diagnostic biomarkers through a combination of morphological classification and immunohistochemical and molecular biology techniques. Although abnormalities in some molecules do not impact the classification of specific tumor subtypes, the treatment and prognosis of patients with tumors with specific molecular abnormalities require further in‐depth study.^1^ The SWItch/Sucrose NonFermentable complex (SWI/SNF) is an ATPase‐dependent chromatin remodeling unit comprising at least 15 protein subunits encoded by nearly 22 genes that regulate transcriptional processes, promote cellular differentiation and play an important role in DNA injury repair. *SMARCA4* is a tumor suppressor gene involved in the catalytic subunit that makes up this complex by encoding the BRG1 protein.^2^ Approximately 10% of non‐small cell lung cancers (NSCLCs) are susceptible to SMARCA4 loss. Loss of SMARCA4 leads to the occurrence of advanced dedifferentiated tumors and increases the incidence of tumor metastasis.^3^ Approximately 83% of patients with SMARCA4‐deficient non‐small cell lung cancer (SMARCA4‐dNSCLC) are at stage IV at the time of discovery, with a median progression‐free survival time of only 30 days.^4^ Although platinum‐based chemotherapy regimens combined with immune checkpoint inhibitors (ICIs) are currently most commonly used in clinical practice, their efficacy is unknown. Nevertheless, it is known that some patients can benefit from first‐line immunotherapy.^5^ Therefore, there is a need to clearly classify NSCLC subtypes by accurate molecular testing to understand the evolution of the condition and select a reasonable treatment modality. In our study, we described the clinicopathological features of 105 SMARCA4‐dNSCLC patients in detail and analyzed the factors affecting their prognosis.

## 2 MATERIALS AND METHODS

### 2.1 Case data and follow‐up

From October 30, 2019, to July 15, 2022, 105 SMARCA4‐dNSCLC patients and 221 SMARCA4‐intact non‐small cell lung cancer (SMARCA4‐iNSCLC) patients were screened out of 1520 NSCLC patients who underwent BRG1 molecular detection by immunohistochemistry (IHC) at the First Affiliated Hospital of Zhengzhou University. The clinical data were collected, including sex, age, smoking history, IHC molecular results, next‐generation sequencing (NGS) results, expression of PD‐L1, tumor‐node‐metastasis (TNM) stage of the primary tumor, treatment regimen, and survival status. The inclusion criteria for SMARCA4‐dNSCLC were pathologically confirmed NSCLC with negative or partially negative expression of BRG1 according to IHC. Two hundred twenty‐one patients with pathologically confirmed BRG1‐positive NSCLC selected using a random sampling method between October 30, 2019 and July 15, 2022 were included in the SMARCA4‐iNSCLC cohort. This study was approved by the Institutional Review Board of the First Affiliated Hospital of Zhengzhou University (2022‐KY‐1394‐002). Informed consent was waived because the study was retrospective. Patients were followed up using medical documents or telephone interviews. The last follow‐up date was October 13, 2022, with a median follow‐up time of 9.0 months. In patients with SMARCA4‐dNSCLC, 48 patients died, and 57 patients were alive at the last follow‐up date.

### 2.2 Genetic testing and IHC analysis

IHC was performed using the EnVision two‐step method to detect BRG1 expression by dilution of BRG1 antibodies (Zhongshan Jinqiao Company). BRG1 expression deletion was defined as a complete loss or significant reduction in nuclear expression in tumor cells compared to strong nuclear expression in background stromal cells or nontumor epithelial cells. NGS was performed using the Human Multigene Mutation Detection Generic Kit, the Human Multigene Mutation Detection Capture Probe, and the Illumina Miniseq sequencing platform. There were two types of customized probe panels for hybridization enrichment, either *SMARCA4* and other tumor‐associated genes (425 preset genes) or probes containing only commonly used tumor‐associated genes (e.g., *EGFR*). All tests were performed in accordance with reagent instructions and laboratory specifications.

### 2.3 Statistical analysis

Data analysis was performed using SPSS 21.0, and the results were plotted in R version 3.2.0 and GraphPad Prism 8.0. Overall survival (OS) refers to the time from the date of pathological diagnosis to the date of the last follow‐up or death. Median overall survival (mOS) refers to the corresponding survival time of patients when the cumulative survival rate is 50%. The *t* test or Wilcoxon test and chi‐square test or Fisher's exact test were used to compare the clinical features of patients with SMARCA4 loss and SMARCA4 intact. Prognostic factors for OS were identified using Cox proportional hazard models. The factors with *p* < 0.2 during univariate Cox analysis were included in the multivariate Cox regression model. *p* < 0.05 was considered statistically significant in multivariate analysis.

## 3 RESULTS

### 3.1 General information

The age of the SMARCA4‐dNSCLC patients ranged from 48 to 83 years old (median 67 years old), the male‐to‐female ratio was 34:1, 77% of the patients had a smoking history, 66% of the patients had stage IV disease, and the tumor size ranged from 0.7 to 12.8 cm (median 4.0 cm). In 60% of patients with SMARCA4‐dNSCLC, the lesions were located in the upper lobe of the lung, and 69% of the SMARCA4‐dNSCLC patients had peripheral lung cancer. A total of 88% of the SMARCA4‐dNSCLC patients had a tumor proliferation index (Ki‐67) ≥30%. These metastatic sites in 69 SMARCA4‐dNSCLC patients with metastases were the bone (32 cases), pleura (27 cases), lungs (24 cases), brain (17 cases), adrenal glands (16 cases), liver (7 cases), pericardium (6 cases), kidney (3 cases), muscle (2 cases), spleen (2 cases), subcutaneous tissue (1 case), throat (1 case), and pelvic cavity (1 case) (some patients had multiple metastases). The pathological types of patients with SMARCA4‐dNSCLC were adenocarcinoma (74%), squamous cell carcinoma (10%), poorly differentiated carcinoma (14%), sarcomatoid carcinoma (1%), and neuroendocrine carcinoma (1%).

The comparison of clinical features between SMARCA4‐dNSCLC and SMARCA4‐iNSCLC is shown in Table 1. SMARCA4‐dNSCLC was significantly associated with older age, male sex, smoking history, larger invasive tumor size, higher tumor proliferation index (Ki‐67), more adrenal metastases, more lymph node metastases, and few *EGFR* mutations (*p* < 0.05). Compared with SMARCA4‐iNSCLC, SMARCA4‐dNSCLC exhibited relatively few squamous cell carcinomas and more poorly differentiated carcinomas (*p* < 0.05).

**TABLE 1:** Clinical features of 105 patients with SMARCA4‐dNSCLC.

Parameters	NSCLC			Parameters	NSCLC		
	SMARCA4 (−) *n* (%)	SMARCA4 (+) *n* (%)	*p*		SMARCA4 (−) *n* (%)	SMARCA4 (+) *n* (%)	*p*
Age, years			<0.01	Brain metastasis			0.94
Median	67	64		Positive	17 (16)	35 (16)	
Range	48–83	26–84		Negative	88 (84)	186 (84)	
Sex			<0.01	Adrenal metastasis			0.02
Male	102 (97)	133 (60)		Positive	16 (15)	15 (7)	
Female	3 (3)	88 (40)		Negative	89 (85)	206 (93)	
Smoking History			<0.01	Other site metastasis			
Ever	81 (77)	84 (38)		Positive	23 (22)	17 (8)	
Never	24 (23)	137 (62)		Negative	82 (78)	204 (92)	
Invasive Tumor Size, cm			<0.01	Stage at presentation			0.37
Median	4.0	3.2		Stage I	11 (10)	38 (17)	
Range	0.7–12.8	0.7–11.1		Stage II	4 (4)	7 (3)	
Ki‐67 (+) (%)			<0.01	Stage III	21 (20)	34 (15)	
≥30%	92 (88)	114 (52)		Stage IV	69 (66)	142 (64)	
<30%	11 (10)	76 (34)		Lymph node metastasis			0.01
Not evaluated	2 (2)	31 (14)		Positive	85 (81)	150 (68)	
PD‐L1 expression			0.95	Negative	20 (19)	71 (32)	
0%–1%	27 (26)	68 (31)		Clinical M stage			0.52
1%–49%	19 (18)	46 (21)		M0	36 (34)	84 (38)	
≥50%	7 (7)	20 (9)		M1	69 (66)	137 (62)	
Not evaluated	52 (50)	87 (39)		*EGFR* mutation			<0.01
Tumor location			0.66	Positive	5 (5)	107 (49)	
Right upper lobe	34 (32)	71 (32)		Negative	57 (54)	74 (33)	
Right middle lobe	5 (5)	21 (10)		Not evaluated	43 (41)	40 (18)	
Right lower lobe	20 (19)	37 (17)		Histology			<0.01
Left upper lobe	29 (28)	55 (25)		Adenocarcinoma	78 (74)	167 (76)	
Left lower lobe	17 (16)	37 (17)		Squamous cell carcinoma	10 (10)	48 (22)	
The location type of the lung			0.59	Poorly differentiated carcinoma	15 (14)	1 (0)	
Central	33 (31)	63 (29)		Sarcomatoid carcinoma	1 (1)	2 (1)	
Peripheral	72 (69)	158 (71)		Neuroendocrine differentiated carcinoma	1 (1)	3 (1)	
Bone metastases			0.42	Treatment	Stage I/Stage II/Stage III/Stage IV		
Positive	32 (30)	58 (26)		Local treatment^a^	1/1/2/2	1/0/0/1	
Negative	73 (70)	163 (74)		Surgery^b^	8/3/6/3	27/5/13/10	
Pleural invasion			0.24	Chemotherapy^c^	0/0/7/31	0/2/10/60	
Positive	27 (26)	71 (32)		Chemotherapy + Immunotherapy^d^	0/0/2/19	1/0/4/17	
Negative	78 (74)	150 (68)		Targeted drugs^e^	0/0/0/2	3/0/4/43	
Intrapulmonary metastasis			0.07	Untreated^f^	2/0/4/12	6/0/3/11	
Positive	24 (23)	72 (33)					
Negative	81 (77)	149 (67)					

^a^ Local treatment included bronchial artery chemoembolization, microwave ablation, and particle implantation.

^b^ Patients who underwent surgery, perhaps with pre‐ or postoperative chemotherapy or radiotherapy.

^c^ Chemotherapy alone regimens included paclitaxel/docetaxel/pemetrexed ± platinum ± antiangiogenic agents.

^d^ Four of all patients treated with immunotherapy received PD‐1 inhibitors alone, and the remaining patients were treated with PD‐1 inhibitors combined with chemotherapeutic drugs.

^e^ Two patients with SMARCA4‐dNSCLC were treated with targeted drugs, one patient received almonertinib alone, and the other patient received ensartinib in combination with chemotherapy. Among the patients with SMARCA4‐iNSCLCs treated with targeted drugs, 23 patients were treated with targeted drugs combined with chemotherapy, and the remaining patients were treated with targeted agents alone.

^f^ Some patients discontinued treatment due to older age, financial difficulties, or severe illness.

### 3.2 Gene mutation types

**FIGURE 1:**
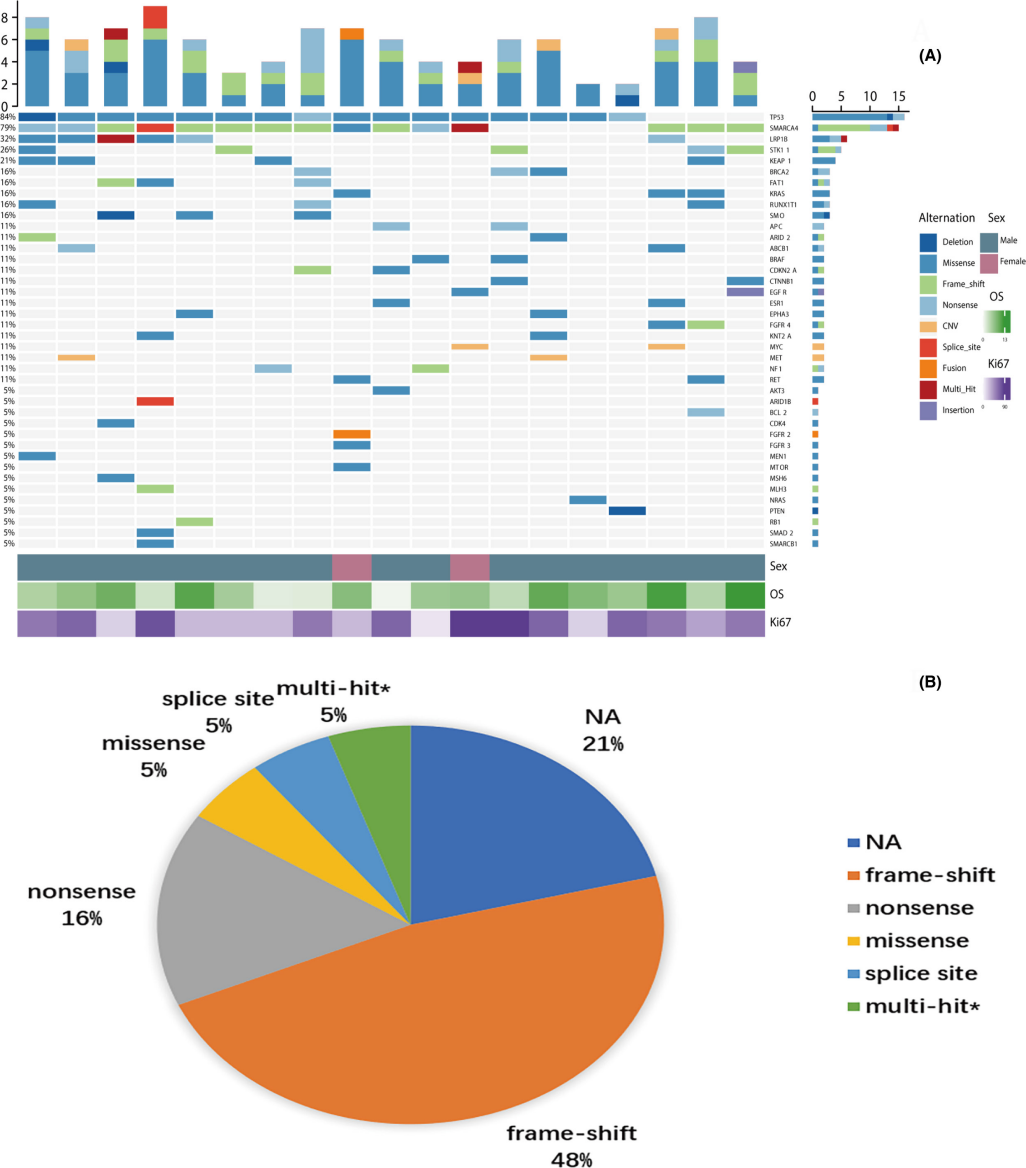
Gene Mutations in 19 SMARCA4‐dNSCLC Patients. (A) Nineteen SMARCA4‐dNSCLC patients underwent NGS to detect concomitant mutated gene status; (B) The pie chart shows *SMARCA4* gene mutation categories for 19 patients. *multi‐hit: *SMARCA4* exon 22, 24 missense mutations and exon 26 nonsense mutation.

*SMARCA4* gene mutations were present in 15 of 19 SMARCA4‐dNSCLC patients who underwent NGS testing with 425 preset genes (including the *SMARCA4* gene). Among the 19 SMARCA4‐dNSCLC patients, 48% had *SMARCA4* frameshift mutations, 21% had no *SMARCA4* mutation, 16% had *SMARCA4* nonsense mutations, 5% had *SMARCA4* missense mutations or splice site mutations, and the other 5% had *SMARCA4* exon 22, 24 missense mutations and exon 26 nonsense mutation. And among the 19 SMARCA4‐dNSCLC patients, 84% had *TP53* mutations, 79% had *SMARCA4* mutations, 32% had *LRP1B* mutations, 26% had *STK11* mutations, and 21% had *KEAP1* mutations. *SMARCA4* mutations most often co‐occurred with alterations in *TP53* (80%), *LRP1B* (40%), *STK11* (27%), *KEAP1* (27%), and *KRAS* (20%). The mean TMB value was 10.43 mutations/Mb. The remaining concomitantly mutated genes are shown in Figure 1.

### 3.3 Therapeutic approach

Among the 105 SMARCA4‐dNSCLC patients, 18 received no treatment, and the rest received the first‐line treatment regimen as follows (Table 1): stage I and II patients underwent local treatment or surgical treatment; among stage III patients, 7 received chemotherapy, 6 received surgery, 2 received local treatment, and 2 received immunotherapy combined with chemotherapy; and among stage IV patients, 31 received chemotherapy, 19 received immunotherapy (immunotherapy combined with chemotherapy in 17, PD‐1 inhibitors alone in 2), 3 received surgery (palliative care), 2 received local treatment, and 2 received targeted drugs. Of the 18 untreated patients, 8 discontinued treatment for economic reasons, 6 received symptomatic treatment due to severe disease, and 4 refused treatment due to their old age (Table S1).

Special cases of targeted therapy were as follows. Two patients with stage IV disease chose targeted drug therapy, including a 62‐year‐old female patient with no smoking history diagnosed with right upper lobe adenocarcinoma with secondary malignant tumors of the brain, adrenal gland, pleura, and lung. Immunohistochemical analysis indicated SMARCA4 (+), thyroid transcription factor‐1 (TTF‐1) (+), and p40(−). Genetic testing revealed an *EGFR* exon 21 L858R missense mutation. Four months after oral administration of osimertinib mesylate tablets, two cycles of bevacizumab combined with pemetrexed and carboplatin chemotherapy were performed due to disease progression. Then, the tumor did not shrink, and the right upper lobe tumor was biopsied again to determine the gene mutation status. The histology revealed poorly differentiated squamous cell carcinoma with SMARCA4 deletion, and IHC indicated AE1/AE3 (+), cytokeratin 7 (CK7) (+), cytokeratin 5/6 (CK5/6) (focal +), p40 (partial +), TTF‐1 (−), napsin A (−), synaptophysin (Syn) (−), SMARCA4 (−), integrase interactor 1 (INI‐1) (+), Ki‐67 (90% +). NGS analysis indicated *EGFR* exon 21 L858R missense mutation, *SMARCA4* exon 22, 24 missense mutations, and exon 26 nonsense mutation, combined *MYC*, *TP53*, and other mutations. Oral administration of almonertinib was initiated. Unfortunately, the patient died with an overall survival time of 0.1 months. Another patient, a 54‐year‐old male with no smoking history, was diagnosed with poorly differentiated adenocarcinoma with SMARCA4 deletion on June 25, 2022. NGS analysis suggested a *ROS1* fusion mutation. He was treated with pemetrexed combined with carboplatin and ensartinib and was alive at the last follow‐up date with an overall survival time of 3.7 months.

### 3.4 Pathological features

Among the 105 SMARCA4‐dNSCLC patients, BRG1 protein was completely absent in 96% and partially absent in 4%. Eighty‐two percent of SMARCA4‐dNSCLC patients were positive for CK7, 86% were negative for CK5/6, 76% were negative for TTF‐1, 79% were negative for p40 and 65% were negative for CD34. Strong positive expression of Sal‐like protein 4 (SALL4) was observed in 23% of SMARCA4‐dNSCLC patients, and the same proportion demonstrated partial positive expression. These pathological features are shown in Table 2.

**TABLE 2:** Pathological features of SMARCA4‐dNSCLC.

IHC (No./total cases)	SMARCA4‐dNSCLC
BRG1	
Loss	96% (101/105)
Partial loss	4% (4/105)
CK7	
Positive	63% (38/60)
Partial positive	18% (11/60)
CK5/6	
Positive	3% (3/93)
Partial positive	11% (10/93)
TTF‐1	
Positive	10% (11/110)
Partial positive	14% (15/110)
p40	
Positive	3% (3/105)
Partial positive	18% (19/105)
CD34	
Positive	17% (4/23)
Partial positive	17% (4/23)
SALL‐4	
Positive	23% (5/22)
Partial positive	23% (5/22)

### 3.5 Survival analysis

**FIGURE 2:**
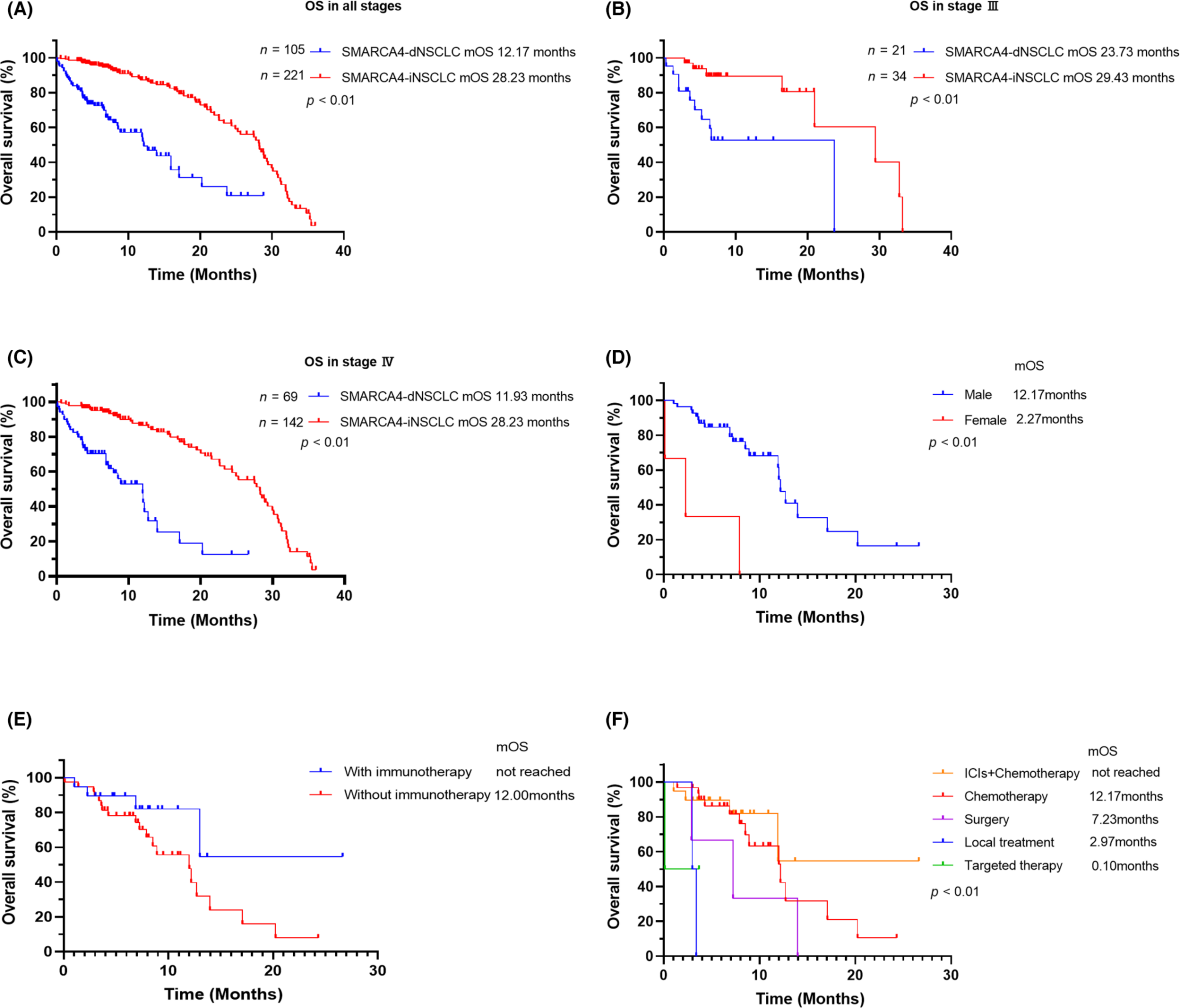
Analysis of Different Kaplan–Meier Curves of SMARCA4‐dNSCLC Patients. (A) OS comparison in patients with all stages SMARCA4‐dNSCLC and SMARCA4‐iNSCLC; (B) OS comparison in patients with stage III SMARCA4‐dNSCLC and SMARCA4‐iNSCLC; (C) OS comparison in patients with stage IV SMARCA4‐dNSCLC and SMARCA4‐iNSCLC; (D) OS comparison of different sexes in patients with stage IV SMARCA4‐dNSCLC; (E) OS comparison of immunotherapy in patients with stage IV SMARCA4‐dNSCLC; (F) OS comparison of different treatment regimens in patients with stage IV SMARCA4‐dNSCLC.

In NSCLC patients, multivariate Cox regression analysis showed SMARCA4 status (HR: 0.27; 95% CI: 0.17–0.45), smoking history (HR: 1.72; 95% CI: 1.11–2.66), and invasive tumor size (HR: 1.11; 95% CI: 1.01–1.23) were independent factors affecting the prognosis of NSCLC, as shown in Table S2. The overall prognosis of patients with SMARCA4‐dNSCLC was poor compared with that of patients with SMARCA4‐iNSCLC, as shown in Figure 2A. The patients were divided into stages I–IV according to the pathological stage. Only patients with stage III (HR: 0.24; 95% CI: 0.09–0.69) and IV (HR: 0.19; 95% CI: 0.11–0.31) SMARCA4‐dNSCLC had a significantly worse prognosis than SMARCA4‐iNSCLC patients, as shown in Figure 2B,C. In patients with SMARCA4‐dNSCLC, the mOS was 12.2 months (95% CI 7.56–16.76), the 1‐year survival rate was 51%, and the 2‐year survival rate was 20%.

Multivariate regression analysis showed that sex (HR: 4.12; 95% CI: 1.03–16.39) and smoking history (HR: 2.29; 95% CI: 1.04–5.02) had significant effects on the survival time of SMARCA4‐dNSCLC patients (Table 3). In patients with SMARCA4‐dNSCLC, who did not have distant metastases (stage I‐III), multivariate Cox regression showed that patients with lymph node stage N2 or N3 (HR: 6.35; 95% CI: 1.07–37.47) had a poor prognosis (Table S3). The Kaplan–Meier curves for patients with SMARCA4‐dNSCLC who had distant metastases (stage IV) and received treatment showed that the mOS had a longer trend in male patients (*p* < 0.01) and patients treated with immunotherapy combined with chemotherapy (*p* = 0.15) (Figure 2C,D), while multivariate Cox regression analysis showed an increased risk of death in female patients (HR: 12.64; 95% CI: 2.53–63.24), as shown in Table 4. The difference in the OS of stage IV patients with different treatment modalities is shown in Figure 2E.

**TABLE 3:** Analysis of prognostic factors in SMARCA4‐dNSCLC patients.

Factor	Univariate analysis			Multivariate analysis		
	HR	95% CI	*p* value	HR	95% CI	*p* value
Clinical features						
Sex (Female/Male)	4.865	1.486–15.930	0.009	4.117	1.034–16.388	0.045
Age (year)	1.043	1.004–1.083	0.028	1.039	0.999–1.080	0.058
History of smoking (+/−)	1.607	0.785–3.289	0.194	2.287	1.041–5.023	0.039
History of drinking (+/−)	1.321	0.674–2.588	0.417			
Ki‐67 (%)	1.008	0.994–1.022	0.253			
PD‐L1	1.003	0.985–1.021	0.745			
Invasive tumor size (cm)	1.074	0.955–1.207	0.233			
Tumor location (Left lobe/Right lobe)	1.155	0.653–2.045	0.620			
Location type (Peripheral/Central)	1.466	0.744–2.888	0.268			
Clinical T stage (T3 + T4/T1 + T2)	1.655	0.924–2.964	0.090	1.077	0.566–2.047	0.822
Clinical N stage (N2 + N3/N0 + N1)	2.103	1.005–4.401	0.049	1.780	0.754–4.205	0.189
Clinical M stage (M1/M0)	1.666	0.884–3.137	0.114	1.604	0.722–3.561	0.246

**TABLE 4:** Analysis of prognostic factors in SMARCA4‐dNSCLC patients with distant metastases (stage IV) receiving treatment.

Factor	Univariate analysis			Multivariate analysis		
	HR	95% CI	*p* value	HR	95% CI	*p* value
Clinical features						
Sex (Female/Male)	8.855	2.443–32.098	0.001	12.640	2.526–63.241	0.002
Age (years)	1.038	0.989–1.088	0.131	0.994	0.943–1.047	0.816
History of smoking (+/−)	1.712	0.637–4.598	0.286			
History of drinking (+/−)	1.516	0.544–4.226	0.427			
Ki‐67 (%)	1.013	0.994–1.032	0.178	1.018	0.998–1.039	0.072
PD‐L1	1.009	0.986–1.031	0.456			
Invasive tumor size (cm)	0.882	0.724–1.075	0.213			
Tumor location (Left lobe/Right lobe)	0.827	0.345–1.981	0.670			
Location type (Peripheral/Central)	1.316	0.457–3.795	0.611			
Clinical T stage (T3 + T4/T1 + T2)	0.890	0.388–2.041	0.783			
Clinical N stage (N2 + N3/N0 + N1)	0.577	0.193–1.724	0.325			
Immunotherapy (YES/NO)	0.453	0.153–1.335	0.151	0.456	0.149–1.403	0.171

*Note*: Immunotherapy*: Most of the immunotherapy in the table refers to chemotherapy combined with PD‐1 inhibitors, and a small number of patients received only PD‐1 inhibitors.

## 4 DISCUSSION

SMARCA4‐dNSCLC was identified by Wong et al. in 2000 for the first time who reported that BRG1 might act as a tumor suppressor and represent a target for tumor cell destruction.^6^ In version 2021 of the WHO classification of thoracic tumors, thoracic SMARCA4‐deficient undifferentiated tumors (SMARCA4‐UT) were mentioned in the pulmonary epithelial tumor section, refocusing attention on SMARCA4. SMARCA4‐dNSCLC, unlike SMARCA4‐UT, is a type of NSCLC that ranges histologically from well‐differentiated malignant tumors to poorly‐differentiated malignant tumors, including solid adenocarcinomas, mucinous adenocarcinomas, acinar or papillary adenocarcinomas, squamous cell carcinomas, large cell carcinomas, rhabdoid morphology tumors, and malignant tumors containing spindle cell or signet ring cell morphology.^2^ This group of cases showed that SMARCA4‐dNSCLC mainly occurred in male smokers, and the median age of onset was approximately 67 years old. Most patients with SMARCA4‐dNSCLC had stage IV disease at diagnosis, with large aggressive tumors, high tumor proliferation indices (Ki‐67), more adrenal metastases, and more lymph node metastases. Additionally, the prognosis is poor because of its invasive clinical behavior.

SMARCA4‐dNSCLC is often accompanied by mutations in genes such as *SMARCA4*, *KRAS*, *STK11*, *TP53*, *KEAP1*, and *LRP1B* and few mutations in genes such as *EGFR*, *ALK*, and *ROS1*.^4^ Adenocarcinoma patients with *SMARCA4*, *TP53*, or *STK11* mutations have been shown to have a lower survival rate.^7^ Genes such as *KRAS*, *STK11*, and *KEAP1* are associated with patients' smoking history, and patients with *LRP1B* mutations tend to have a higher TMB.^8^ Mutations in the *STK11* and *KEAP1* are associated with resistance to chemotherapy and immunotherapy, respectively.^9^ Among these mutated genes, the types of mutations in the *SMARCA4* are divided into two categories: class I mutations, including truncating mutations (frameshift and nonsense mutations), gene fusions, and homozygous deletions, and class II mutations, including missense mutations.^10^ Mutations in *SMARCA4* result in loss of BRG1 protein, but NGS testing for *SMARCA4* gene mutation status is not required if negative BRG1 protein expression is confirmed by IHC. For example, of the 19 patients examined by NGS in this study, 4 patients had no *SMARCA4* gene mutation, but BRG1 loss was detected by IHC, which was considered to result from structural variants in the intronic region of the gene, microRNA (miR‐155)‐mediated posttranscriptional repression or mutations in other SWI/SNF family proteins, especially mutations in the BAF subunit ARID1A.^2^, ^11^ Loss of BRG1 is associated with class I mutations in the *SMARCA4* gene, and patients with class I mutations have lower survival rates than those with class II mutations; however, interestingly, patients with class I mutations respond better to ICIs,^10^ and class II mutations have also been shown to be deleterious.

Patients with SMARCA4‐dNSCLC had high Ki‐67 indices, more than 50% in 59% of patients and more than 30% in 88% of patients, and large tumors, demonstrating the rapid proliferation of this type of tumor. The mean TMB value of patients with SMARCA4‐dNSCLC who underwent NGS testing was 10.43 mutations/Mb, and PD‐L1 >1% of patients with SMARCA4‐dNSCLC accounted for 49%. As biomarkers that can predict immune efficacy, PD‐L1 and TMB prove that ICIs may play an important role in treating SMARCA4‐dNSCLC. Naito et al. demonstrated that NSCLC patients with loss of SWI/SNF complex subunit expression (SMARCA4, SMARCA2, ARID1A, or ARID1B) were more likely to have aggressive clinicopathologic features, positive PD‐L1 status, and a high TMB.^12^

In rare case reports of SMARCA4‐dNSCLC, IHC features include CK7 (+), hepatocyte paraffin 1 (HepPar‐1) (+), TTF‐1 (−), claudin‐4 (+), p40/p63 (−), napsin (−), CK5/6 (−), and negative expression of neuroendocrine markers; except for SALL4, most SMARCA4‐dNSCLC are negative for stem cell markers, such as SRY‐box 2 (SOX2) and CD34.^13^, ^14^, ^15^ These immunochemical profiles were consistent with the patients in the current study. IHC molecular features are helpful to differentiate from other similar tumors. Unlike SMARCA4‐dNSCLC, classical lung adenocarcinomas are mostly TTF‐1 positive expression and HepPar‐1 negative expression, SMARCA4‐UTs are usually positive for stem cell markers and negative for epithelial markers.

Available evidence suggests that SMARCA4‐dNSCLC is a type of NSCLC with a poor prognosis. Some researchers have proposed that the median survival time of 11 patients with stage IV disease was 4.4 months, significantly shorter than that of patients with stage II/III disease.^2^ Bell et al. proposed that low SMARCA4 expression was associated with decreased OS.^16^ In our study, patients with SMARCA4‐dNSCLC had significantly shorter OS than SMARCA4‐iNSCLC patients. For the first time, we studied factors influencing the prognosis of 105 patients with SMARCA4‐dNSCLC. Our research proved that sex and smoking history were associated with the prognosis of SMARCA4‐dNSCLC. NSCLC patients have similar findings. In a prospective study, Sheikh et al. found a reduced risk of death and disease progression in NSCLC patients who quit smoking.^17^ Hu et al. demonstrated that smoking was a risk factor for patients with advanced NSCLC in China.^18^ There is no doubt that the different TNM stages of lung cancers have a significant impact on the OS of patients. In patients with SMARCA4‐dNSCLC who did not have distant metastases, we found that patients with stage N2 or N3 lymph node metastases had a poor prognosis. In a multivariate model of SMARCA4‐dNSCLC patients treated in stage IV, patient prognosis also differed significantly based on sex. Our study showed that female patients with SMARCA4‐dNSCLC were at higher risk of death. It is undeniable that the number of female SMARCA4‐dNSCLC patients is very small, which may cause differences in the results.

At present, there is no clear recommended regimen for treating SMARCA4‐dNSCLC. Because patients with advanced SMARCA4‐dNSCLC have few common locus mutations, chemotherapy or immunotherapy is often used in these patients. However, in the current group of patients, two patients with SMARCA4‐dNSCLC had common genetic locus mutations, and the tumor‐type transformation in one of them was curious. This patient's tumor transformed in situ from adenocarcinoma to poorly differentiated squamous cell carcinoma with SMARCA4 loss, and the current reasonable hypothesis is a branched evolutionary model with cells of common origin,^19^, ^20^ i.e., there is a precursor cell at baseline that can transform into another phenotype in response to TKI treatment. Our finding of early resistance to osimertinib in this patient and the emergence of mutations at novel genetic loci (*SMARCA4* gene) following resistance and the presence of preexisting mutations at *EGFR* exon 21 support this notion.^21^

Existing treatment options for SMARCA4‐dNSCLC include drugs that target oxidative phosphorylation processes and inhibit SMARCA2, EZH2, ATR, CDK4, or CDK6 molecules.^22^, ^23^, ^24^, ^25^ Additionally, cisplatin and vinorelbine can be used as effective regimens for resectable SMARCA4‐dNSCLC in stage IB–II.^16^ SMARCA4‐dNSCLC other than stage I has a high risk of relapse following surgery, and immunotherapy is effective.^26^ In a study by Shinno et al. on ICIs in SMARCA4‐deficient thoracic tumors, the response rate to ICIs was 42%, and the progression‐free survival time with ICIs was significantly longer with the first‐line regimen than with the second‐line or later treatment; additionally, TMB could be used to predict patients who would benefit from ICIs.^5^ Our study found that patients with advanced SMARCA4‐dNSCLC had a longer mOS with ICI treatment combined with chemotherapy. However, for patients with advanced SMARCA4‐dNSCLC, there was no significant difference in OS between patients who received immunotherapy and those who did not. This may indicate that we need a longer follow‐up period. Aurora kinase A (AURKA) is required for mitotic spindle assembly; the AURKA inhibitor VX‐680 is also being evaluated to treat SMARCA4‐dNSCLC.^27^

This study was a retrospective study, and there were two limitations. (1) Treatment regimens were classified according to the first‐line selection, and the differences in progression‐free survival between different treatment schemes were not calculated. (2) Since this was a retrospective study, PD‐L1, NGS, and some specific IHC molecules were not detected in each patient. It is difficult to analyze the relationship between TMB or PD‐L1 and immunotherapy efficacy in detail.

In summary, by analyzing the clinical features, gene mutation information, and factors affecting the prognosis of 105 SMARCA4‐dNSCLC patients, we demonstrated that SMARCA4‐dNSCLC is a type of tumor distinct from SMARCA4‐iNSCLC, with poor prognosis and high invasiveness. Patients treated with immunotherapy plus chemotherapy had a longer mOS than those treated with other therapies. Nevertheless, it is imperative that specific types of tumors be identified early and that novel treatments be explored.

## AUTHOR CONTRIBUTIONS

**Xiyue Liang:** Conceptualization (equal); writing – original draft (equal). **Xianzheng Gao:** Resources (equal); supervision (equal). **Feng Wang:** Conceptualization (equal); resources (equal); supervision (equal). **Shenglei Li:** Resources (equal); supervision (equal). **Yashu Zhou:** Data curation (equal); resources (equal); software (equal). **Peng Guo:** Software (equal); validation (equal). **Yuanyuan Meng:** Supervision (equal); visualization (equal). **Taiying Lu:** Formal analysis (equal); funding acquisition (equal); supervision (equal); writing – review and editing (equal).

## FUNDING INFORMATION

This research was supported by the 2020 Medical Science and Technology Research (Key) Project of Henan Province (SBGJ202002068) and the National Key Research and Development Program of China (2018YFC1705102).

## CONFLICT OF INTEREST STATEMENT

The authors declare that the research was conducted in the absence of any commercial or financial relationships that could be construed as a potential conflict of interest.

## ETHICS STATEMENT

This was an observational study. The study involving human participants was reviewed and approved by the Institutional Review Board of the First Affiliated Hospital of Zhengzhou University (2022‐KY‐1394‐002). Written informed consent for participation was not required for this study in accordance with the national legislation and institutional requirements.

## Supporting information


Table S1.
Table S2.
Table S3.

## Data Availability

The original contributions presented in the study are included in the article material. Further inquiries can be directed to the corresponding author.
