# Supplementary material for: Clinical characteristics and prognostic analysis of SMARCA4‐deficient non‐small cell lung cancer
Source: Cancer Med. 2023 May 15;12(13):14171–82. doi: 10.1002/cam4.6083 (PMC10358186; doi:10.1002/cam4.6083)
Supplement: Supplementary file 1 — Table S1. Table S2. Table S3. [file CAM4-12-14171-s001.docx]

Table 1. General Information of 18 Untreated Patients

| ID | Sex | Age  (years) | History of smoking | Invasive tumor size(cm) | Stage | Reason for rejection | Status | OS  (months) |
| --- | --- | --- | --- | --- | --- | --- | --- | --- |
| 1 | Male | 63 | Ever | 6.16 | ⅣB | Economic issues | Died | 6.87 |
| 2 | Male | 67 | Never | 3.50 | ⅠB | Economic issues | Alive | 5.70 |
| 3 | Male | 78 | Ever | 8.70 | ⅣA | Old age | Alive | 5.37 |
| 4 | Male | 68 | Ever | 10.00 | ⅢB | Economic issues | Died | 4.33 |
| 5 | Male | 73 | Ever | 2.10 | ⅣB | Economic issues | Died | 3.97 |
| 6 | Male | 79 | Ever | 7.30 | ⅣB | Old age | Died | 3.60 |
| 7 | Male | 72 | Ever | 3.20 | ⅢA | Economic issues | Died | 2.07 |
| 8 | Male | 77 | Ever | 7.00 | ⅢC | Old age | Died | 2.07 |
| 9 | Male | 59 | Ever | 10.26 | ⅣA | Economic issues | Died | 1.87 |
| 10 | Male | 70 | Ever | 4.70 | ⅣB | Economic issues | Died | 1.73 |
| 11 | Male | 52 | Ever | 2.39 | ⅣA | Economic issues | Died | 1.53 |
| 12 | Male | 64 | Ever | 3.60 | ⅢA | Severe condition | Died | 1.30 |
| 13 | Male | 74 | Ever | 3.10 | ⅠB | Severe condition | Died | 1.30 |
| 14 | Male | 67 | Ever | 3.20 | ⅣB | Severe condition | Died | 1.03 |
| 15 | Male | 71 | Ever | 12.80 | ⅣA | Severe condition | Died | 0.80 |
| 16 | Male | 53 | Ever | 7.00 | ⅣB | Severe condition | Died | 0.40 |
| 17 | Male | 81 | Ever | 5.47 | ⅣB | Old age | Died | 0.33 |
| 18 | Male | 76 | Ever | 11.00 | ⅣB | Severe condition | Died | 0.10 |

Table 2. Analysis of Prognostic Factors in NSCLC Patients

|  |  |  | Univariate analysis | | | Multivariate analysis | | |
| --- | --- | --- | --- | --- | --- | --- | --- | --- |
| Factor |  |  | HR | 95%CI | *P* value | HR | 95%CI | *P* value |
| Clinical features | Sex | (Female/Male） | 0.743 | 0.491-1.125 | 0.161 |  |  |  |
|  | Age | (year) | 1.019 | 1.001-1.038 | 0.038 | 1.008 | 0.986-1.029 | 0.478 |
|  | History of smoking | (+/-) | 0.743 | 0.491-1.125 | 0.001 | 1.718 | 1.109-2.661 | 0.015 |
|  | History of drinking | (+/-) | 1.169 | 0.733-1.865 | 0.513 |  |  |  |
|  | Ki-67 | (%) | 0.012 | 1.004-1.020 | 0.002 | 0.999 | 0.989-1.008 | 0.754 |
|  | PD-L1 |  | 0.995 | 0.983-1.006 | 0.371 |  |  |  |
|  | Invasive tumor size | (cm) | 0.012 | 1.004-1.020 | 0.002 | 1.114 | 1.012-1.227 | 0.028 |
|  | Tumor location | (Left lobe/Right lobe) | 1.132 | 0.786-1.631 | 0.505 |  |  |  |
|  | SMARCA4 Status | (+/-) | 0.234 | 0.154-0.356 | <0.001 | 0.274 | 0.168-0.445 | <0.001 |
|  | location type | (Peripheral/Central) | 0.812 | 0.537-1.228 | 0.323 |  |  |  |
|  | TNM staging | (Ⅳ/Ⅲ/Ⅱ/Ⅰ) |  |  | 0.138 |  |  |  |

Table 3. Analysis of Prognostic Factors in SMARCA4-dNSCLC Patients with No Distant Metastases (stage Ⅰ-Ⅲ) Receiving Treatment

|  |  |  | Univariate analysis | | | Multivariate analysis | | |
| --- | --- | --- | --- | --- | --- | --- | --- | --- |
| Factor |  |  | HR | 95%CI | *P* value | HR | 95%CI | *P* value |
| Clinical features | Age | (years) | 1.074 | 0.953-1.212 | 0.242 |  |  |  |
|  | History of smoking | (+/-) | 1.299 | 0.155-10.864 | 0.809 |  |  |  |
|  | History of drinking | (+/-) | 1.306 | 0.238-7.165 | 0.759 |  |  |  |
|  | Ki-67 | (%) | 0.990 | 0.952-1.029 | 0.603 |  |  |  |
|  | PD-L1 |  | 0.977 | 0.874-1.093 | 0.688 |  |  |  |
|  | Invasive tumor size | (cm) | 1.070 | 0.709-1.615 | 0.748 |  |  |  |
|  | Tumor location | (Left lobe/  Right lobe) | 7.754 | 0.953-63.096 | 0.056 | 2.944 | 0.296-29.231 | 0.357 |
|  | location type | (Peripheral/Central) | 5.901 | 0.724-48.125 | 0.097 | 8.281 | 0.649-105.613 | 0.104 |
|  | Clinical T stage | (T3+T4/ T1+T2) | 1.223 | 0.218-6.851 | 0.819 |  |  |  |
|  | Clinical N stage | (N2+N3/N0+N1) | 4.156 | 0.973-17.744 | 0.054 | 6.345 | 1.074-37.474 | 0.041 |
|  | Surgery | (YES / NO) | 0.330 | 0.082-1.335 | 0.120 | 0.538 | 0.110-2.625 | 0.444 |
